# A Systematic Review of Submetatarsal Fat Pad Augmentation for the Treatment and Prevention of Diabetes‐Related Foot Ulceration

**DOI:** 10.1002/jfa2.70064

**Published:** 2025-08-31

**Authors:** Christopher Ashmore, Jagdeep Virdee, Peter Culmer, Jennifer Edwards, Heidi Siddle, James Warren, David Russell

**Affiliations:** ^1^ University of Leeds Faculty of Medicine and Health University of Leeds Leeds UK; ^2^ Royal Hallamshire Hospital Sheffield Teaching Hospitals NHS Foundation Trust Sheffield UK; ^3^ University of Leeds School of Mechanical Engineering University of Leeds Leeds UK; ^4^ Faculty of Biological Sciences Institute of Medical and Biological Engineering University of Leeds Leeds UK; ^5^ Leeds Institute of Rheumatic and Musculoskeletal Medicine University of Leeds Leeds UK; ^6^ Leeds Institute of Clinical Trials Research University of Leeds Leeds UK

**Keywords:** adipose tissue, allografts, autografts, diabetes, diabetic foot, diabetic foot ulcer, fat pad

## Abstract

**Background:**

Diabetes‐related foot ulceration (DFU) represents a significant and increasing cause of morbidity and economic burden to health services. Surgical offloading has shown great effectiveness in the prevention and healing of DFU. The objective of this review is to assess the effectiveness of submetatarsal plantar fat pad modulation in preventing DFU and to characterise the different biomaterials used to this end.

**Methods:**

The study was registered on PROSPERO. A search strategy of the PubMed, CINAHL and Cochrane biomedical databases was conducted. Any study which explored the modulation of the plantar submetatarsal fat pad for the prevention or treatment of DFU in adults was included. The main outcome was the occurrence of ulceration following intervention.

**Results:**

Of the 3162 retrieved studies, 10 studies met inclusion criteria, describing outcomes for 76 participants with 112 ulcers or pre‐ulcerative areas. Four studies report results of injectable liquid silicone in 55 participants, four studies included the use of an acellular allograft in eight participants, two studies included autolipotransplantation in 11 participants and one study reports on the use of injectable collagen in two participants. Only one randomised control trial was identified while the remainder of the studies were observational, case‐series, or case‐reports. The overall ulcer occurrence was 27/112 over an average follow‐up of 32.4 months.

**Discussion:**

While plantar fat pad modulation shows promise as a surgical offloading strategy for DFU, insufficient high‐quality trial data preclude meaningful interpretation of its merits. This is further complicated by heterogeneity in the biomaterial employed for modulation.

## Graphical abstract

This systematic review evaluates the effectiveness of submetatarsal fat pad augmentation for diabetic foot ulcer prevention. While current studies suggest benefit, methodological quality is poor and biomaterials are heterogeneous. Identification and optimisation of a suitable biomaterial are needed before meaningful controlled trials can establish efficacy and position fat pad modulation as a standard therapeutic approach.

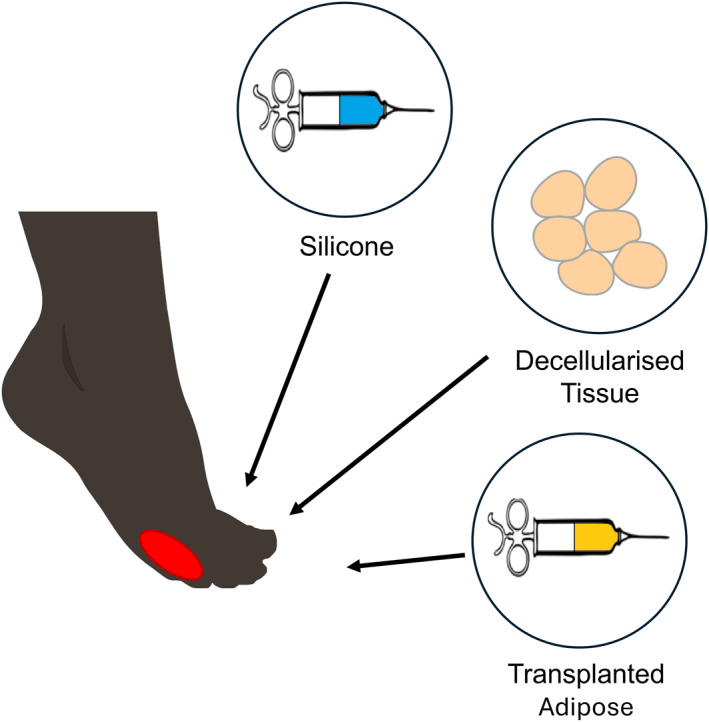

**Abbreviations**
ALT: Autolipotransplantation
DFU: Diabetic Foot Ulceration
DM: Diabetes Mellitus
IWGDF: International Working Group of the Diabetic Foot
JBI: Joanna Briggs Institute
MT: Metatarsal
MTPJ: Metatarsophalangeal Joint

## 1 Introduction

Globally, an estimated 500 million people are living with diabetes mellitus (DM) [1], of which five million reside within the United Kingdom [2]. Diabetes‐related foot ulceration (DFU) affects up to a third of people with DM and has a recurrence rate of 65% at 5 years, globally [3]. Associated with significant morbidity, DFU complications include infection, loss of limb and loss of life. The 5‐year survival following the development of a DFU is 30.5% and the mortality following a major limb amputation is 56.6% [4]. As a result, DFU represents a source of both considerable morbidity and significant healthcare costs, estimated at close to £1billion annually to the National Health Service in England alone [5].

DFU is a multifactorial disease process which results in a final common pathway of dermal breakdown and exposure of underlying deep tissues. Advanced glycation end products contribute to the development of sensory, motor and autonomic neuropathy resulting in foot deformity and the loss of protective sensation [6]. This is often compounded by comorbid peripheral arterial and microvascular disease, particularly in the context of a precipitating injury [7].

The plantar fat pad consists of fixed, septated adipose tissue, permeated by neurovascular bundles which terminate in the dermis [8]. Parallel collagen fibres and elastin form the septal wall, providing fibrous attachments between the skin and deep tissues [9], whilst also arranging tightly‐packed adipose cells into load‐dissipating curved formations [10]. Collectively, these structures lie superficial to the underlying metatarsals (MT), flexor tendons and associated nerves, acting as a shock absorber to offset load‐associated dermal injury [9]. In the context of DM, the honeycombed septated adipose structure is replaced with nodular, irregular type III collagen and broken elastin fibres [11]. Degeneration, in conjunction with atrophy or displacement [9], results in the alteration of pedal biomechanics, leading to elevated peak plantar pressures and likely contributing to ulceration [12]. Reconstruction of the plantar fat pad has therefore been pursued as a modality to reduce the rate of plantar ulceration [13, 14, 15, 16].

Previous reviews in this area have employed non‐systematic methodologies to collate literature for the description of liquid silicone to reduce plantar pressure [17], autologous fat grafting in the non‐diabetic foot [18], skin‐grafting and coverage of DFU [19], pathological mechanisms of plantar fat pad migration [9] and the use of dermal fillers for the treatment of metatarsalgia [20]. To date, there is no systematic interrogation of the literature to evaluate the effectiveness of plantar fat augmentation in the treatment and prevention of DFU. Accordingly, this review has sought to collate and analyse existing evidence to answer the research question: ‘Does plantar fat pad modulation reduce the occurrence of DFU?’

## 2 Materials and Methods

A project protocol was registered on PROSPERO prior to the completion of searches. Three electronic biomedical databases (PubMed, CINAHL, Cochrane) along with two further pre‐publication registries (MedRxiv, PROSPERO) were interrogated in November 2023 using a defined search strategy (Appendix A). The search strategy included the Medical Subject Heading terms ‘Allografts’, ‘Autografts’, ‘Fat Pads’, ‘Diabetic Foot’ and ‘Foot Ulcer’. Results were transferred to Rayyan.ai [21] for manual de‐duplication and screening. Two independent reviewers used a predefined protocol for screening of the results at the abstract level. Conflicts were to be resolved by a third independent reviewer, who was not required. Articles of potential interest were then screened by both reviewers at the full‐text level.

### 2.1 Inclusion/Exclusion Criteria

Studies were included if they examined any plantar fat pad intervention in the context of diabetic foot disease in adults. Fat pad interventions limited to the calcaneum or for non‐diabetic atrophy or foot pain were excluded, as were articles which examined topical or intra‐lesional treatments of active foot ulcers. Randomised control trials, cohort studies, observational studies, case series and case reports were included while review articles, opinion articles, abstract‐only publications and non‐English language articles were excluded. There were no limitations on the date of publication.

### 2.2 Data Items

The primary outcome was the occurrence of DFU. This was defined as the development of new ulceration at any site in the foot or the failure to heal ulceration following a fat pad intervention. This outcome was chosen as it captures the treatment strategies for pre‐ulcerative lesions, active ulcers and at‐risk sites of prior ulceration. Secondary outcomes included callus burden, changes in peak plantar pressures and complications following intervention.

Data extraction was achieved through the use of standardised tables. Data capture included study type, population characteristics, and details of the fat pad intervention, ulcer activity and location, both quantitative and qualitative outcomes for plantar pressures, complications, callus burden and changes in plantar thickness. Where studies included a heterogenous indication, only the diabetes‐related subgroup was extracted. Quality assessment was performed using the Cochrane Risk of Bias Score 2 Checklist for randomised control trials [22], the Joanna Briggs Institute (JBI) checklist for case series [23] or the Murad tool for case reports [24]. These were chosen on the basis of their specificity to the methodological design labels, wide availability of resources and the derivation of the tools from the perspective of intervention efficacy. In particular, tools sometimes employed for the assessment of methodological quality of case reports and case series lack specificity and hence possess less discriminatory power [25]. Articles were reviewed by two independent reviewers using the relevant quality assessment tool and scored with reference to their subjective utility in this systematic review. Where uncertainty was identified within a quality assessment domain, no point was awarded.

### 2.3 Data Reporting

A meta‐analysis of odds ratios to compare combined interventions to usual care for the development of DFU was planned. A secondary analysis contrasting outcomes for different modalities of intervention was also considered. However, due to a relative paucity of controlled trials, considerable data heterogeneity and inconsistency of reporting, meta‐analysis was not attempted in accordance with the Cochrane Handbook for Systematic Reviews of Interventions [26]. Instead, reporting of results took the form of narrative review, adhering to the Synthesis Without Meta‐analysis guidelines [27]. Studies were grouped by the nature of the intervention to the fat pad due to the expectation that different modalities of fat pad augmentation would be associated with different risk and benefit profiles.

## 3 Results

**FIGURE 1:**
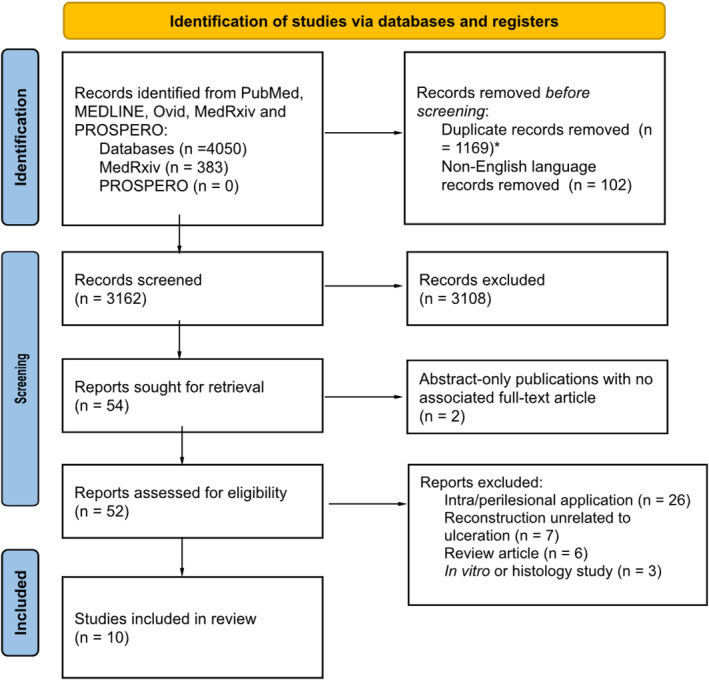
PRISMA flow diagram of study selection process. Flowchart illustrating the systematic review process, including database search results, study screening, eligibility assessment and final inclusion criteria. *De‐duplication assisted by integrated text‐matching feature of Rayyan.ai; Text matching of 100% was removed automatically (*n* = 102); remaining high‐probability duplicates were reviewed manually prior to removal (*n* = 1076). Following this process, no additional duplicates were identified.

Following the removal of duplicates, 3162 articles were identified through our search strategy. Of these, 10 studies met the criteria for inclusion (Figure 1). Of the 10 studies, only one was a randomised control trial and a second included study was a 2‐year follow‐up observational study from this trial. The remaining studies were case reports or case series, reporting retrospective outcomes of patients who had undergone fat pad augmentation. Four studies reported results from injectable liquid silicone, three from acellular allograft, one from Autolipotransplantation (ALT), one from injectable collagen and one combining acellular adipose injection with ALT (Table 1).

**TABLE 1:** Summary of studies on submetatarsal fat pad augmentation for diabetes‐related foot ulceration.

Study	Authors	Year	Study type	Intervention	Participants undergoing intervention (controls)	Feet (right)	Individuals with active ulceration (ulcers)	Individuals with pre‐ulcerative callus (sites)	Individuals with healed ulcers (ulcers)	Av. Length of follow‐up (months)	Individuals with ulcer occurrence at conclusion (DFU), individuals with DFU %	Ulcers/yr.	Change in plantar thickness (mm)	Callus improved
Efficacy of injected liquid silicone in the diabetic foot to reduce risk factors for ulceration: a randomized double‐blind placebo‐controlled trial [13].	van Schie et al.	2000	Randomised control trial	Injectable liquid silicone	14 (14)	Not reported	0	14	0	12	3 (3), 21.4%	3	1.8	Trend towards improvement
The effect of silicone injections in the diabetic foot on peak plantar pressure and plantar tissue thickness: a 2‐year follow‐up [28]	van Schie et al.	2002	Follow‐up observational study	Injectable liquid silicone	11 (5)	Not reported	0	11	0	24	2 (8), 18.2%	4	1.1	Not reported
The fluid silicone prosthesis^a^ ^,^ [29]	Balkin	1984	Case series	Injectable liquid silicone	11	14 (6)	1 (2)	3 (5)	7 (11)	138	5 (6), 45.5%	0.52	Not reported	81%
Injectable silicone and the diabetic foot: a 25‐year report^a^ ^,^ [15]	Balkin and Kaplan	1991	Case series	Injectable liquid silicone	30	Not reported	Not reported (3)	8 (16)	Not reported (36)	75.6	Not reported (9)	0.95	Not reported	100%
Augmentation of atrophic plantar soft tissue with an acellular dermal allograft: a series review^a^ ^,^ [16]	Rocchio	2009	Case series/case report	Sheet acellular dermal allograft	5	Not reported	3 (3)	1	1	27	0 (0), 0%	0	Preserved	Not reported
Early clinical experience with the use of a new allograft adipose matrix for foot fat pad restoration^a^ ^,^ [30]	Schoenhaus Gold	2023	Case series	Injectable acellular adipose allograft	2	Not reported	0	1 (1)	1 (1)	20	0 (0), 0%	0	Not reported	100%
Tissue augmentation with allograft adipose matrix for the diabetic foot in remission [31]	Shahin et al.	2017	Case report	Injectable acellular adipose allograft	1	1 (0)	0	0	1 (1)	4	0 (0), 0%	0	Not reported	100%
Remission strategies with fat grafting to prevent recurrent pedal ulceration^a^ ^,^ [32]	Kress et al.	2023	Case series	ALT or injectable acellular dermal allograft	10	11 (6)	7	3	0	9.3	0 (0), 0%	0	Not reported	Not reported
Plantar fat grafting and tendon balancing for the diabetic foot ulcer in remission [33]	Luu et al.	2016	Case report	ALT (following tendon transfer)	1	1 (1)	0	1 (1)	0	1.5	0 (0), 0%	0	Preserved	100%
Use of injectable collagen to treat chronic diabetic foot ulcers [34]	Skorman	1987	Case series	Injectable collagen	2	3 (2)	1 (1)	2 (2)	0	13	1 (1), 50%	0.92	Not reported	Not reported

*Note:* Overview of included studies inclusive of details on study design and clinical outcomes. Categorised by intervention type, ordered by hierarchy of evidence then alphabetically by author.

^a^ Articles in which a relevant subpopulation was extracted.

### 3.1 Clinical Outcomes

Seventy‐six participants with 17 active ulcers and 95 pre‐ulcerative lesions underwent plantar fat pad augmentation for diabetes‐related foot disease. Following intervention, the average length of follow‐up was 32.4 months, during which there were 27 ulcer episodes, of which 11 were pre‐existent ulcers which had failed to heal prior to fat pad intervention. All studies (10/10) showed positive direction of effect of plantar fat pad modulation. Plantar thickness, where measured, was preserved or increased following intervention [13, 16, 28, 33] and the frequency or appearance of pre‐ulcerative callus was improved in 92.2% of patients [29, 30, 31, 33]. Complications reported include two hallux and three lesser toe minor amputations and one major lower limb amputation.

### 3.2 Risk of Bias

Results from the quality assessment are shown in Tables 2, 3, 4. Assessment using the Murad tool (Table 2) identified adequate ascertainment of intervention and outcome for all three case reports. However, there were concerns about the representativeness of selected patients to the population presenting with DFU in all cases. In two reports, the included patients were young (aged 31–38) [33, 34] while the third lacked sufficient detail to ascertain generalisability [31]. In another instance, a participant underwent multiple procedures in the form of ALT and tendon transfer [33], obfuscating the precipitant for healing. A challenge rechallenge phenomenon was only reported in one instance [34]. The JBI tool for case series (Table 3) identified similar issues with case selection, as only two articles reported inclusion or exclusion criteria that influenced decisions for fat pad augmentation [16, 29]. Clinical and demographic information reporting was generally limited and no robust statistical analysis was completed in any of the articles. However, three reviews report outcomes for all consecutive patients over a defined time‐period [16, 29, 32], of which one was judged to have clearly reported relevant clinical outcomes [16]. Assessment made with the Cochrane Risk of Bias 2 Tool (Table 4) identified a low risk of bias for selection, allocation and reporting in an initial randomised control trial of injectable liquid silicone against usual care [13] but did identify some risk of bias in the domains of missing data and outcome reporting in a follow‐up study, largely due to loss of participants from the control arm at 48 months [28].

**TABLE 2:** Quality assessment of case reports using the Murad tool.

Case report	Murad tool								Total
Study	Representative patient	Exposure adequately ascertained	Outcome adequately ascertained	Alternative causes excluded	Challenge/rechallenge phenomenon	Dose–response effect	Adequate follow‐up	Sufficient detail for replication	
Luu et al. 2016		*	*	*				*	4/8
Skorman 1987		*	*	*	*		*	*	6/8
Shahin et al. 2017		*	*				*	*	4/8

*Note:* Evaluation of methodological quality for case reports based on eight key criteria, as shown.

**TABLE 3:** Quality assessment of case series using the JBI critical appraisal tool.

Case series	JBI tool for case series										Total
Study	Clear inclusion criteria	Standardised measurement of condition	Methods for identification of the condition	Consecutive patients reported	Complete inclusion	Participant demographic reporting	Clear clinical information reporting	Clear outcome reporting	Reporting of clinic demographics	Appropriate statistical analysis	
Balkin 1984	*		*	*	*						4/10
Balkin and Kaplan 1991			*				*				2/10
Kress et al. 2023			*	*	*	*	*				5/10
Rocchio 2009	*		*	*	*	*		*			6/10
Schoenhaus Gold 2023		*	*				*				3/10

*Note:* Assessment of case series quality using the JBI criteria based on 10 key criteria, as shown.

**TABLE 4:** Quality assessment of randomized controlled trials using the Cochrane Risk of Bias 2 tool.

Randomised control trial	Cochrane Risk of Bias 2 score					
Study	Randomisation process	Deviations from intended interventions	Missing outcome data	Measurement of the outcome	Selection of the reported result	Total
van Schie et al. 2000	Low	Low	Low	Low	Low	Low risk of bias
van Schie et al. 2002	Low	Low	Some concerns	Low	Some concerns	Some concerns

*Note:* Evaluation of randomized controlled trials (RCTs) based on risk of bias in five key domains, as shown.

### 3.3 Liquid Silicone

Four studies explored the use of injectable pre‐cured liquid silicone for plantar fat pad augmentation (Table 5). Balkin [29] reports on the use of polydimethylsiloxane (liquid silicone) in the feet of 740 individuals. This included a subgroup of 11 people with DM undergoing treatment for 5 pre‐ulcerative lesions, 11 sites of healed plantar ulceration and 2 active plantar ulcers, with 0.5–10 mL of liquid silicone given in 4–15 injections. Over a mean follow‐up of 11.5 years, 2 active ulcers failed to improve and 4 plantar ulcers occurred. Improvement in callus was noted in 13 of 16 cases, reducing the necessary frequency of consultation. In an updated retrospective series, Balkin and Kaplan [15] subsequently expanded this work to report outcomes for patients with injectable liquid silicone in an extended cohort of 30 people with DM undergoing treatment for 3 (29 plantar, 7 digital) healed ulcers, 16 painless keratoses and 3 active plantar ulcers. This demonstrated a plantar ulcer occurrence in 9 out of 32 treated plantar sites over a mean length of follow‐up of 6.3 years. Of the 3 reported ulcers injected prior to healing, all failed to heal at 2 years. In addition, 7 lesser toe digital ulcers, treated with injectable liquid silicone into the toe after healing, did not recur over a mean follow‐up of 7.3 years. None of an additional 16 treated pre‐ulcerative keratoses, without prior ulceration, recurred or ulcerated.

**TABLE 5:** Patient demographics and intervention details for injectable liquid silicone studies.

	Participants	Intervention	Sex F/M	Mean age	Duration of diabetes	ABPI
Van Schie et al. 2002 [28]	14	11	1/10	55.2	13.2	1.2
Van Schie et al. 2000 [13]	28	14	8/20	56.6	12.8	1.2
Balkin and Kaplan 1991 [15]	30	30	15/15	59.9	14.2	—
Balkin 1984 [29]	11	11	8/3	59.9	—	—

*Note:* Comparison of study populations treated with injectable liquid silicone, including sample size, sex distribution, age, diabetes duration and ankle‐brachial pressure index (ABPI), where available.

**FIGURE 2:**
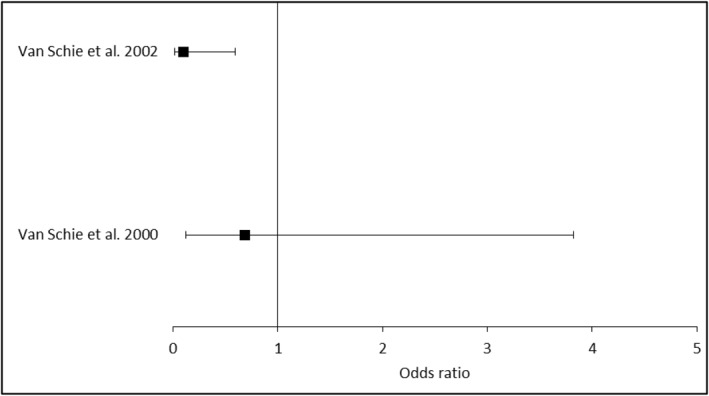
Forest plot of ulcer occurrence following injectable silicone treatment. Brief meta‐analysis of ulcer occurrence rates comparing injectable liquid silicone intervention versus control, showing odds ratios and confidence intervals for each study.

Van Schie et al. [13] undertook the only randomised control trial of liquid silicone, recruiting 28 people with DM and neuropathy with pre‐ulcerative lesions. 14 participants received 34 0.2 mL injections of silicone, contrasting 14 control participants who received 28 saline injections. This demonstrated a modest reduction in ulcer occurrence (3 vs. 4, *p* = 0.676) at 1 year. However, the number of patients who developed DFU was significantly lower following silicone injection at 2‐year follow‐up [28] (2 vs. 9, OR = 0.09, *p* = 0.012, Figure 2). Corresponding increases in ultrasound‐measured plantar thickness at 3‐month (1.8 vs. 0.08 mm, *p* < 0.05) were largely retained at 2 years (1.1 mm vs. −0.1 mm, *p* = 0.001). However, reductions in mean peak plantar pressure associated with silicone injection at 3 months (−232 kPa vs. −25 kPa, *p* < 0.05) slowly diminished over the course of follow‐up, with eventual loss of significance at 2‐year (−23.5 vs. 36.9 kPa, *p* = 0.89). The authors identified that silicone‐mediated reductions in the pressure time integral at 12 months (−0.71 vs. 0.44 kPa) were preserved at 24 months (−0.25 vs. 0.64 kPa), providing a potential mechanism for the improved outcomes. In addition, a visual scoring system devised to grade callus showed a trend towards improvement in the treated cohort [13].

### 3.4 Injectable Collagen

Only Skorman [34] reports on the use of injectable collagen in two cases of active diabetic foot ulceration managed with Keragen (collagen) Implant [35]. This biomaterial is a bovine xenograft consisting of solubilised and purified subdermal collagen which is subjected to glutaraldehyde cross‐linking for stabilisation [36]. In the case reports, a 31‐year‐old male patient received a 0.75 mL injection of collagen beneath a left fifth plantar MT ulcer, complicated by local infection necessitating oral antimicrobials. Bilateral plantar hallux ulcers and a right fifth MT ulcer were initially left as untreated as control lesions. Subsequent interval treatment of the now healed right hallux ulcer with collagen injection was performed following re‐epithelialisation. This enabled healing at the left fifth MT and prevented recurrence at either implanted site. In contrast, there was relative stasis of the remaining control lesions, complicated by eventual left hallux and right fifth ray amputation. The second patient received implantation under an ulcer at the right first metatarsophalangeal joint (MTPJ) resulting in healing at 4 months with no local complications. Overall, both patients achieved healing following collagen‐injection mediated offloading of the active DFU.

### 3.5 Autolipotransplantation

Two publications report the results of ALT, a process through which distant subcutaneous adipose, usually in the abdomen or thigh, is aspirated and injected into the foot. Typically, this involves an incision at the donor site for vacuum‐assisted aspiration of adipose and infiltrated saline through a custom syringe, separation of adipose from infiltrated saline using various techniques and re‐infiltration of harvested adipose into the recipient site via a stab incision [37]. A case report by Luu et al. [33] describes abdominal lipoaspiration with ALT to the plantar surface of the fifth MT following tendon transfer for fat pad wasting and keratosis. At 6 weeks, preserved plantar tissue thickness and no occurrence of DFU was observed. This contradicts findings in non‐diabetic populations whereby initial increases in plantar thickness following ALT were not retained at 2‐month [38]. Kress et al. [32] have also recently reported outcomes following plantar fat grafting in which 15 individuals received ALT (11) or acellular adipose allograft (4) (Leneva, MTF biologics) for recurrent plantar DFU following healing. A subpopulation of eight people with DM underwent ALT of 13.9 +/− 8.7 mL for seven sites of active ulceration and three sites of prior ulceration. Over an average of 7 months of follow‐up, there were no cases of ulcer occurrence and no reported complications at either the donor or recipient site.

### 3.6 Decellularised Tissue

Various decellularised allogeneic scaffolds have been explored for plantar tissue augmentation (Table 6). Rocchio [16] reports a consecutive series of patients undergoing 26 instances of augmentation of the plantar fat pad with an acellular dermal allograft (Graftjacket Regenerative Tissue Matrix, Wright), of which five people had DM. Three individuals had active DFU, one was in remission and one had pre‐ulcerative keratosis. Following rehydration in saline, the human dermal graft was implanted using an open ‘parachute’ technique, in which traction sutures positioned at four corners of the graft are driven through the deep tissues of the wound to exit through the skin. This permits uniform, flush positioning of the graft at the base of the wound. Of the population with DM, all achieved healing at the site of implantation and DFU. No occurrence of DFU over an average follow‐up of 6 months was reported. Overall, the study reported an average increase in plantar tissue thickness of 7.03 mm with one complication in which a patient suffered wound infection with dehiscence, requiring debridement and antimicrobials.

**TABLE 6:** Properties and sources of acellular biomaterials used in fat pad augmentation.

Study	Individuals undergoing intervention	Material	Tissue	Form	Product	Manufacturer
Rocchio 2009 [16]	5	Decellularised dermal tissue	Human	Tissue matrix	Graftjacket MaxForce	Wright medical
Shahin et al. 2017 [31]	1	Decellularised adipose tissue	Human	Injectable	—	MTF biologics
Kress et al. 2023 [32]	2	Decellularised adipose tissue	Human	Injectable	Leneva	MTF biologics
Shoenhaus Gold 2023 [30]	3	Decellularised adipose tissue	Human	Injectable	Leneva	MTF biologics

*Note:* Description of acellular biomaterial types used in plantar fat pad augmentation, including tissue origin, processing method and commercial manufacturer.

An alternative biomaterial is acellular adipose. Kress et al. [32] report two cases of fat pad augmentation by way of an injectable allogeneic acellular adipose (Leneva, MTF biologics) implantation in their multi‐modality plantar intervention case series, in which they also explored ALT. Both had forefoot ulceration which healed following the procedure with a mean healing time of 33 days. There were no reported complications and no reported ulcer recurrence at follow‐up over an average of 9.3 months. Similarly, Schoenhaus Gold [30] reports experience with this material in the treatment of two pre‐ulcerative (Wagner grade 0) lesions and one superficial (Wagner grade 1) ulcer, a subpopulation from a case series of patients undergoing treatment for plantar fat pad atrophy. This resulted in healing of the active ulcer and no recurrence in any patients at follow‐up ranging from 3 to 14 months. There was also an unquantified reduction of hyperkeratotic lesions in all patients. While no quantification of plantar thickness for this subpopulation is available, subsequent work by the author exploring fat pad reconstruction in 18 patients for non‐diabetic pathologies failed to show retention of plantar thickness at 24 weeks, despite improvements in pain scores, function and peak pressures [30].

## 4 Discussion

### 4.1 Surgical Offloading

Peripheral sensory neuropathy and foot deformity contribute to high peak pressures, callus formation and eventual ulceration in the diabetic foot [39]. Offloading orthoses are a mainstay treatment of the neuropathic diabetic foot, redistributing pressure away from problem areas, improving healing of DFU and reducing recurrence [40]. However, adherence with offloading devices is frequently poor and certain devices may pose an unacceptable fall risk within a vulnerable demographic [41]. Surgical offloading seeks to provide similar benefit by directly correcting deformities which contribute to ulceration. Techniques include digital tenotomy, Achilles tendon lengthening, surgical exostectomy, first MTPJ arthroplasty, stabilisation procedures and podoplastic foot reconstruction [42]. The demonstrated benefit of flexor tenotomy in the treatment of pre‐ulcerative callus and active DFU at the digital apices [43, 44] has led to its inclusion in the 2023 International Working Group of the Diabetic Foot (IWGDF) guidelines [45]. Similarly, the effectiveness of Achilles tendon lengthening for the treatment of recalcitrant forefoot ulceration for the healing of forefoot DFU, prevention of recurrence and mitigation of peak plantar pressures [46, 47] along with lower‐quality evidence supporting first MTPJ arthroplasty [48] have led to an IWGDF recommendation for their use in DFU failing to improve with orthotic offloading alone. To date, there is no recommendation for fat pad modulation in the prevention or treatment of DFU. Our review did not find sufficient high‐quality evidence to support the use of fat pad modulation for DFU prevention as a standard of care, despite the described positive direction of effect, due to lack of control data available in the identified studies. However, the identified literature suggests that this is an area of great promise for future work, particularly given successes in the small scale randomised control trial [13, 28].

### 4.2 Prosthetic Materials

Pre‐cured liquid silicone represents a viscous, biologically inert material with the potential to modulate plantar tissue and hence prevent DFU [17, 29, 49]. The included studies in our review demonstrate benefit in the increasing of plantar thickness, reductions in plantar pressures and decreased ulceration. In addition to diabetic indications, plantar silicone injection has been used to treat plantar fasciitis [50] and keratomas with mixed results [51, 52]. However, concern exists about the potential complications from silicone injection. Local effects such as infection, inflammatory response or local migration are perhaps of less concern than particulate uptake by histiocytes and distant migration to regional lymph nodes [17]. While no visceral deposition or systemic sequelae have been described in 1585 individuals undergoing silicone injection in the foot [53], the de facto ban of silicone as a medical device in the wake of scandals arising from its use in breast augmentation prostheses has effectively halted further research in this avenue [54]. Because of the initial promising work in mitigating DFU, there has been a notable movement towards the exploration of alternative biomaterials.

### 4.3 Autolipotransplantation

ALT addresses several challenges posed by prosthetic implants. Autologous transfer of tissue reduces concerns surrounding biocompatibility while providing a biosimilar material for plantar cushioning. Adipose tissue improves skin quality [55], possesses mechanical properties compatible with energy dissipation [56], and, unlike acellular products, also provides a rich source of adipose‐derived stem cells [57]. Accordingly, this method represents the restoration of plantar fat with a biomaterial possessing desirable characteristics and a logical underpinning for the prevention and treatment of DFU. The identified literature in this review generally supports the effectiveness of ALT, however this was limited and of low quality. Further, there are several challenges with the adoption of ALT as the default technique for fat pad modulation. The nature of adipose harvesting prior to transplantation means that complications and challenges must be considered at both the donor and recipient site. Donor site problems include diabetes‐induced poor quality adipose [58], inadequate tissue for harvest, haematomas, infections and liposuction deformities. Recipient site problems include the additional risks of significant swelling and the risk of fat embolism syndrome from inadvertent intravascular injection [55, 59]. Further, there is concern about the longevity of transplanted adipose in the foot. Results from non‐diabetes related reconstructions show significantly diminished volume after 1 year [38]. This could perhaps be explained by the loss of the adipose‐supporting microenvironment through harvest and processing. Further, the regenerative effect of adipose‐derived stem cells, while promising, have not consistently translated into an observed benefit for the treatment of active DFU [60, 61]. These factors, when combined with the need for specialist equipment and relatively lengthy procedures [55], suggest that a more robust biomaterial of ready availability for fat pad modulation may be desirable.

### 4.4 Decellularised Tissues for Fat Pad Modulation

Decellularisation is an increasingly popular area for medical device development. By removing cellular components, the remnant acellular tissue should present a relatively biologically inert material which, following implantation, has the potential to act as a scaffold for recellularisation and regeneration [62]. Various mechanical and chemical techniques have been used in the generation of acellular tissue and no single method has emerged as an industry standard [63]. Further challenges in acellular biomaterial manufacture are dependent on the choice of tissue origin. For example, porcine acellular adipose would require more thorough removal of specific xenogeneic antigens whereas human acellular adipose may be limited by tissue availability [64]. While adipose may seem an obvious target, our review has identified low‐quality evidence of benefit in the use of both acellular human adipose and acellular human dermis for plantar fat pad modulation. While cases of apparent successes have been reported in this review, no single technique or product has emerged for fat pad modulation.

### 4.5 Limitations

This study utilised a systematic search strategy in the identification of relevant literature and reported findings in accordance with the Cochrane Handbook for Systematic Reviews of Interventions. However, there were a number of limitations which should be highlighted. Heterogenous patient populations, interventions and reporting limit meaningful comparison of studies. A dearth of control data meant that studies were at high risk of reporting bias and rendering meta‐analysis impossible. Finally, the literature search was limited to English language publications and valuable non‐English resources may have been missed.

## 5 Conclusion

While insufficient evidence exists to support fat pad modulation for the treatment or prevention of DFU, early clinical studies have elicited some promising results. Silicone has perhaps justifiably fallen out of favour and heterogeneity exists in the implementation of ALT and insertion of acellular biomaterials. The IDEAL framework describes the process of surgical innovation, through which new surgical technologies should progress before becoming the standard of care [65]. The stages of surgical evolution, numbered sequentially, are described qualitatively as idea, development, exploration, assessment and long‐term surveillance. Applying the case of fat pad modulation to this framework, the environment remains at product development and pre‐analytical refinement (stage 1–2), requiring identification, development and further characterisation of the optimal biomaterial. Both ALT and acellular techniques are yet to achieve consensus on a single device or mode of delivery. Future work should seek to explore and refine material options, identifying a product which is biocompatible, long‐lasting and possessing suitable mechanical properties. There also exists a need to further our understanding of the optimal method and location for implantation. Similarly, the mode of harvest and delivery for ALT could be compared, refined and long‐term retention observed. Only once these techniques have been optimised can comparative trials of efficacy to standard of care be meaningfully achieved.

## Author Contributions

**Christopher Ashmore:** conceptualization, methodology, formal analysis, data curation, writing – original draft, writing – review and editing, project administration. **Jagdeep Virdee:** data curation, writing – review and editing. **Peter Culmer:** conceptualization, supervision, writing – review and editing. **Jennifer Edwards:** conceptualization, supervision, writing – review and editing. **Heidi Siddle:** conceptualization, supervision, writing – review and editing. **David Russell:** conceptualization, supervision, writing – review and editing.

## Conflicts of Interest

The authors declare no conflicts of interest.

## Data Availability

The data that support the findings of this study are available from the corresponding author upon reasonable request.

## Appendix A Search Strategy

1. Autologous OR allogeneic OR autograft* OR Allograft* OR inject*
2. ALLOGRAFTS/
3. AUTOGRAFTS/
4. Sub Total ‐ 1 OR 2 OR 3
5. ‘Fat pad*’ OR Fat OR lipid OR lipo* OR adipo* OR ulcer*
6. FAT PAD/
7. FAT PADS/
8. Sub Total 5 OR 6 OR 7
9. inject* OR filler OR reconstruct* OR modulat* OR augment* OR restor*
10. Sub Total ‐ 4 AND 8 AND 9
11. Foot OR plantar OR submetatarsal OR metatarsal OR sub‐metatarsal OR sole OR ‘diabetic foot’ OR ‘diabetic feet’
12. HEEL/
13. FOOT/
14. DIABETIC FOOT/
15. DIABETIC FEET/
16. FOOT ULCER/
17. FOOT ULCER, DIABETIC/
18. Sub Total – 11 OR 12 OR 13 OR 14 OR 15 OR 16 OR 17
19. Final Total – 10 AND 18
